# Dynamic landscape of long noncoding RNAs during leaf aging in *Arabidopsis*

**DOI:** 10.3389/fpls.2022.1068163

**Published:** 2022-12-01

**Authors:** Jung Yeon Kim, Juhyeon Lee, Myeong Hoon Kang, Tran Thi My Trang, Jusung Lee, Heeho Lee, Hyobin Jeong, Pyung Ok Lim

**Affiliations:** ^1^ Department of New Biology, Daegu Gyeongbuk Institute of Science and Technology (DGIST), Daegu, South Korea; ^2^ Genome Biology Unit, European Molecular Biology Laboratory (EMBL), Meyerhofstraße 1, Heidelberg, Germany; ^3^ Department of Life Science, College of Natural Sciences, Hanyang University, Seoul, South Korea

**Keywords:** leaf senescence, transcriptome (RNA-seq), Arabidopsis, long noncoding RNA, RNA-RNA interaction

## Abstract

Leaf senescence, the last stage of leaf development, is essential for whole-plant fitness as it marks the relocation of nutrients from senescing leaves to reproductive or other developing organs. Temporally coordinated physiological and functional changes along leaf aging are fine-tuned by a highly regulated genetic program involving multi-layered regulatory mechanisms. Long noncoding RNAs (lncRNAs) are newly emerging as hidden players in many biological processes; however, their contribution to leaf senescence has been largely unknown. Here, we performed comprehensive analyses of RNA-seq data representing all developmental stages of leaves to determine the genome-wide lncRNA landscape along leaf aging. A total of 771 lncRNAs, including 232 unannotated lncRNAs, were identified. Time-course analysis revealed 446 among 771 developmental age-related lncRNAs (AR-lncRNAs). Intriguingly, the expression of AR-lncRNAs was regulated more dynamically in senescing leaves than in growing leaves, revealing the relevant contribution of these lncRNAs to leaf senescence. Further analyses enabled us to infer the function of lncRNAs, based on their interacting miRNA or mRNA partners. We considered functionally diverse lncRNAs including antisense lncRNAs (which regulate overlapping protein-coding genes), competitive endogenous RNAs (ceRNAs; which regulate paired mRNAs using miRNAs as anchors), and mRNA-interacting lncRNAs (which affect the stability of mRNAs). Furthermore, we experimentally validated the senescence regulatory function of three novel AR-lncRNAs including one antisense lncRNA and two mRNA-interacting lncRNAs through molecular and phenotypic analyses. Our study provides a valuable resource of AR-lncRNAs and potential regulatory networks that link the function of coding mRNA and AR-lncRNAs. Together, our results reveal AR-lncRNAs as important elements in the leaf senescence process.

## Introduction

A leaf is a representative organ encompassing the fundamental characteristics of a plant. During the lifespan of a leaf, a series of physiological and functional shifts are observed, which end with senescence and death (Lim et al., 2007). At the early leaf developmental stages, the photosynthetic machinery is assembled *via* the biogenesis of chloroplasts and the synthesis of photosynthetic pigments, which in turn contribute to plant growth. After the maturation stage, leaves undergo organ-level senescence, which involves the orderly disassembly of subcellular organelles and macromolecules and the concomitant relocation of hydrolyzed molecules to actively growing organs such as developing seeds (for successful reproduction in annuals) or storage organs such as stems or roots (for the preparation of the next generation in perennials). Thus, despite its degenerative features, leaf senescence is critical for ensuring optimal offspring production and enhancing plant survival (Lim et al., 2007; Sasi et al., 2022).

Leaf senescence is triggered by an innate developmental program. However, this process is substantially affected by internal factors such as reproduction as well as external factors such as abiotic and biotic stresses. To ensure an integrated response to these internal/external factors, leaf senescence is tightly controlled by an intertwined network of developmental- or stress-associated pathways over time (Woo et al., 2019; Zhang et al., 2021). Attempts have been made to dissect the molecular mechanisms underlying leaf senescence through the identification of senescence-altered mutants and the functional characterization of senescence regulatory genes in *Arabidopsis* as well as in agriculturally important crops, revealing dozens of key regulatory molecules including transcription factors, epigenetic regulators, regulatory microRNAs (miRNAs), protein-modifying molecules, and small secretory peptides, thus expanding our knowledge of how leaf senescence is fine-tuned at the chromatin, transcriptional, post-transcriptional, translational, and post-translational levels (Woo et al., 2019; Zhang et al., 2022). More recently, the temporal dynamics of age-related regulatory networks and molecular mechanisms linking environmental signals with innate senescence pathways have been further explored using multi-omics technologies, together with computational biology tools and extensive biochemical and molecular genetic analyses (Woo et al., 2016; Lyu et al., 2017; Kim et al., 2018).

Previous transcriptome analyses, which mainly focused on protein-coding genes, revealed a detailed chronology of leaf senescence-associated physiological processes, highlighting the transcriptome as a significant molecular signature of leaf senescence (Breeze et al., 2011; Woo et al., 2016) Although these studies are worthy to infer key regulatory elements and pathways, the analyses remain limited to unraveling the hidden layers of leaf senescence-related gene regulatory networks.

Long noncoding RNAs (lncRNAs) are long transcripts (>150 nt) with poor protein-coding potential (<50 amino acids) and are emerging as important modulators of gene expression in diverse biological processes in animals and plants (Dinger et al., 2008; Chen et al., 2020; Statello et al., 2021). The genomic origins and biogenesis processes vary widely among lncRNAs, ranging from intergenic regions, introns of annotated genes, or to the antisense strand of neighboring protein-coding genes (referred to as natural antisense transcripts [NATs]). The lncRNAs are a functionally heterogeneous group of RNA molecules that regulate gene expression by interacting with specific DNAs, RNAs, or proteins through *in cis* or *in trans* mechanisms involving chromatin remodeling (Heo and Sung, 2011), RNA processing (Bardou et al., 2014), RNA stabilization (Ma et al., 2021a), and translational regulation (Jabnoune et al., 2013). The lncRNAs also act as decoys of miRNA or RNA-binding proteins (Seo et al., 2019; Liu et al., 2022), generate miRNAs (Augoff et al., 2012), or are translated into small open reading frames (sORFs) (Romero-Barrios et al., 2018).

Genome-wide transcriptome analyses in plants revealed thousands of lncRNAs, which are differentially expressed in response to abiotic and biotic stresses. However, only limited numbers of plant lncRNAs have been functionally analyzed. For example, *COLD INDUCED LONG ANTISENSE INTRAGENIC RNA* (*COOLAIR*) and *COLD ASSISTED INTRONIC NONCODING RNA* (*COLDAIR*) are involved in vernalization-mediated regulation of flowering (Heo and Sung, 2011); *ELF18-INDUCED LONG-NONCODING RNA1* (*ELENA1*) regulates the expression of *PATHOGENESIS-RELATED1* (*PR1*) gene, which encodes a key plant immunity related protein (Seo et al., 2019).; *INDUCED BY PHOSPHATE STARVATION1* (*IPS1*) regulates phosphate homeostasis as an endogenous target mimic (eTM) of *miR399* (Franco-zorrilla et al., 2007); *HIDDEN TREASURE 1* (*HID1*) affects photomorphogenesis by regulating the expression of the *PHYTOCHROME-INTERACTING FACTOR 3* (*PIF3*) gene (Wang et al., 2014); *AUXIN REGULATED PROMOTER LOOP* (*APOLO*) regulates *PINOID* (*PID*) expression by modulating chromosome loop dynamics, thereby affecting auxin signaling (Ariel et al., 2014). These results indicate that lncRNAs serve as crucial regulators of plant development and stress responses. Recently, the importance of lncRNAs in leaf senescence has been also explored in tomato and rice (Huang et al., 2021; Li et al., 2022). However, their roles in the regulation of leaf senescence still remain poorly understood. One of the major obstacles that inhibit mechanistic studies of leaf senescence-related lncRNAs is the lack of a genome-wide systematic analysis of lncRNAs.

In this study, we performed comprehensive analyses of RNA-seq data collected from *Arabidopsis thaliana* leaves at different developmental stages, leading to the identification of age-related lncRNAs (AR-lncRNAs). Multiple types of analyses were conducted including the characterization of AR-lncRNAs and prediction of their potential target genes by linking the functions of protein-coding RNAs to those of AR-lncRNAs. Knockout mutations of three AR-lncRNAs resulted in altered leaf senescence phenotypes, validating the regulatory role of these AR-lncRNAs in senescence process. Our results will serve as a useful resource and framework for further functional and mechanistic studies to reveal the detailed regulatory role of lncRNAs in *Arabidopsis* leaf senescence.

## Materials and methods

### Data source

The reference genome sequence and transcriptome annotation GTF files of *Arabidopsis thaliana* were downloaded from The Arabidopsis Information Resource (TAIR10.47). The previously published strand-specific total RNA-seq and small RNA (sRNA)-seq data derived from the leaves of *Arabidopsis thaliana* ecotype Columbia (Col-0) (Woo et al., 2016) were used in this study.

### Identification of novel lncRNAs in *Arabidopsis*

The TruSeq adapter sequences were removed using Trimmomatic to obtain clean reads (average Phred quality score ≥ 20), which were then aligned to the *Arabidopsis* reference genome (TAIR10.47) using TopHat2, with a default parameter (mismatch ≤ 2 nt) (Kim et al., 2006a; Bolger et al., 2014). To predict novel transcripts, mapped reads from each bam file were assembled using Cufflinks with the -M parameter (Trapnell et al., 2010). The resultant GTF file was merged with the annotated lncRNAs from TAIR10.47 using cuffmerge. Read counts were then generated using HTseq-count, and expression levels were estimated as transcripts per million (TPM) (Anders et al., 2015). To select *Arabidopsis* lncRNAs, only transcripts with Cufflinks class codes ‘u’ (intergenic transcripts), ‘x’ (exonic overlap with reference sequence on the opposite strand), and ‘i’ (transcripts entirely within the intron) were retained. Then, short transcripts (<150 nt) and low-abundance transcripts (maximum TPM [TPMmax] < 1) were removed. Unannotated transcripts were named using the Cufflinks annotation (XLOC_).

### lncRNA characterization and sORF detection

Small RNA and their precursors were predicted based on the previously published small RNA-seq data of aging leaves (Axtell, 2013b; Woo et al., 2016) using Shortstack with default parameters. Then, the ribosome footprint sequencing (Ribo-seq) data of the leaves of 3-week-old plants (Lukoszek et al., 2016) were used to predict the ribosome-associated lncRNAs (ribo-lncRNAs). Subsequently, putative translated sORFs were predicted by calculating the ribosome release score (RRS), which indicates whether the ribosome footprint decreases after the termination codon of sORFs (≥30 nt, 10 amino acids) (Guttman et al., 2014; Bazin et al., 2017).

### Identification and analysis of differentially expressed genes

Differentially expressed transcripts were identified using the DESeq2 method (Love et al., 2014). Enrichment analysis of Gene Ontology (GO) terms was performed using DAVID v6.8 (Huang et al., 2008).

### Prediction of RNA–RNA interactions

Interaction energies between AR-lncRNAs and mRNAs were calculated from the FASTA file of TAIR10.47 using RIBLAST 1.1.1 (Fukunaga and Hamada, 2017a). with the following thresholds: interaction energy < -16 kcal/mol, and interaction length ≥ 15 nt. Pearson correlation coefficients of the predicted AR-lncRNA and mRNA pairs detected throughout the leaf lifespan were calculated using the average TPM values.

### Plant materials and growth conditions

*Arabidopsis thaliana* ecotype Columbia (Col-0) is the wildtype strain for all mutants. Plants were grown in an environmentally controlled growth room (Korea Instruments, Korea) at 22℃ under 16h light:8h dark photoperiod and photosynthetic photon flux density of 130μmol m^-2^s^-1^. The Arabidopsis transfer DNA (T-DNA) insertion lines, SALK_100875 (*at5g01595*), SALK_151843 (*atfer1*) SALK_124431C (*at1g33415*), and SALK_135316 (*at2g14878*) were obtained from Arabidopsis Biological Resource Center. The genotype of each mutant line was confirmed by PCR-based genotyping.

### Leaf senescence assay

For developmental leaf senescence, the third and fourth leaves of each plant, at the indicated age, were harvested at 4 to 5h after light-on. For dark-induced senescence, the 14-d-old third and fourth leaves of each plant were detached and floated upside down on 3mM MES buffer (pH 5.7) in 24-well plates, which were completely wrapped with aluminum foil. The photochemical efficiency (F_v_/F_m_) ratio of photosystem II was measured by a Walz IMAGING-PAM machine.

### RNA isolation and quantitative RT-PCR analysis

Total RNA was extracted from third and fourth rosette leaves were extracted with TRIzol and treated with DNase I (Ambion). cDNA was synthesized using the ImPromII™ system (Promega) reverse transcription kit following the manufacturer’s instruction. The extracted cDNA was used for semi-quantitative and quantitative real time Polymerase chain reaction (RT-PCR and qRT-PCR). qRT-PCR analysis was carried out to determine the gene expression levels (CFX96 system, Bio-Rad). Transcript abundances of target genes were analyzed by the comparative threshold method, with *ACTIN2* (*AT3G18780*) as the internal control. For visualization of amplified cDNA band in RT-PCR, 10µl of the amplified product was run in 0.8% agarose gel containing ethidium bromide.

## Results

### Genome-wide identification of lncRNAs in *Arabidopsis* leaves

**Figure 1:**
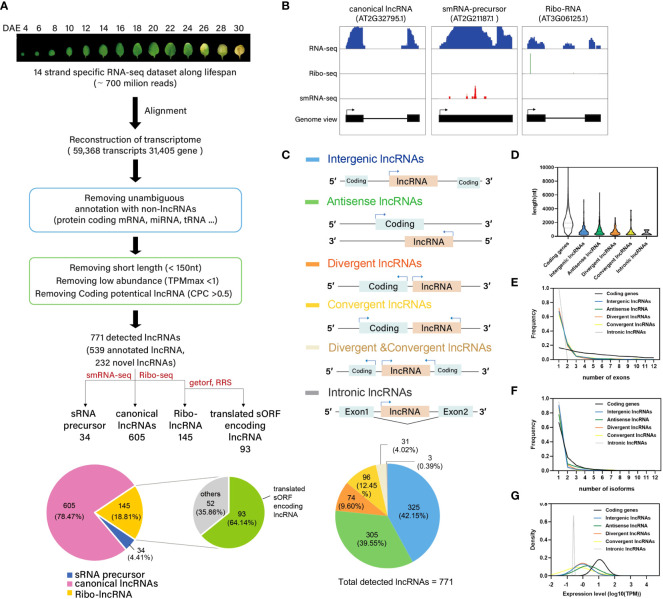
Genome-wide identification of lncRNA in *Arabidopsis*. **(A)** Pipeline for the systematic identification of lncRNAs in *Arabidopsis*. Note that there are 13 detected lncRNAs which were belonging to both sRNA precursor and Ribo-lncRNA. **(B)** Normalized read coverage of representative lncRNAs in each category, as measured by RNA-seq, sRNA-seq, and ribo-seq. **(C)** Classification of lncRNAs based on their genomic location. **(D)** Violin plot showing the length distribution of lncRNAs. **(E)** Frequency line plot showing the distribution of exon numbers in different lncRNAs. **(F)** Frequency line plot showing the distribution of isoform numbers of different lncRNAs. **(G)** Distribution curve of average expression levels of lncRNAs.

Previously, we generated multidimensional transcriptome data of 4-d- to 30-d-old *Arabidopsis* leaves, and characterized the regulatory features of leaf senescence (Woo et al., 2016) (**Supplementary Data 1**). In this study, we re-analyzed our previously generated RNA-seq dataset to assess the role of lncRNAs in the development of leaf organs. A total of 700 million reads were aligned against TAIR10 using TopHat2 (Kim et al., 2006a), and the mapped reads were assembled using Cufflinks (Trapnell et al., 2010). This led to the identification of 59,368 unique transcripts, corresponding to 31,405 gene loci. Among the 59,368 unique transcripts, 50,465 were previously annotated as protein-coding transcripts or as noncoding RNAs such as housekeeping RNAs (e.g., tRNAs, rRNAs, small nuclear RNAs, and small nucleolar RNAs) and miRNA precursors. In addition to these 50,565 transcripts, 4,815 short transcripts (length < 150 nt) and 3,262 low-abundance transcripts (TPMmax < 1) were excluded from the dataset of unique transcripts. Subsequently, 54 transcripts showing protein-coding potential, as determined by the Coding Potential Calculator (CPC) (Kang et al., 2017), were further eliminated. Thus, 771 lncRNAs were identified, of which 539 were previously annotated and 232 were novel (**Figure 1A**, **Supplementary Data 2**).

Based on their predicted functions, the 771 lncRNAs were classified into the following three categories: 1) ribo-lncRNAs, lncRNAs that potentially encode an sORF or are associated with the stability and translation of their cognate mRNAs *in trans*; 2) sRNA precursors, lncRNAs that generate precursors of small RNAs such as miRNAs and *trans*-acting or phased small interfering RNAs (tasiRNAs/phasiRNAs); and 3) other canonical lncRNAs not included in the first two categories (**Figure 1A**). Ribo-lncRNAs (145/771 [18.78%]) were predicted using ribo-seq data generated from the leaves of 3-week-old *Arabidopsis* plants (Lukoszek et al., 2016). Translated sORFs are often hidden among ribo-lncRNAs (93/145 [64.14%]). Potential sORFs encoding > 10 amino acids from ribo-lncRNAs were recognized using two analytics: RRS, which evaluates the decrease in ribosome footprint number after termination codons (Bazin et al., 2017); and getorf, which finds and outputs the sequence of ORFs in nucleotide sequences (Rice et al., 2000). This analysis allows to identify putative lncRNA-encoded sORFs with RRS ≥ 0.9 (29). The remaining ribo-lncRNAs might be involved in the stabilization and translation of their cognate mRNAs or in the *trans*-regulation of mRNAs. LncRNAs capable of generating the precursors of 21–22-nt long sRNAs (34/771 [4.40%]) were predicted based on the small RNA-seq data (Axtell, 2013a; Woo et al., 2016) (**Figure 1A**). Furthermore, we confirmed the RNA-seq read coverage of representative lncRNAs in each category (ribo-lncRNAs, sRNA precursors, and canonical lncRNAs) using gene viewer (**Figure 1B**).

In addition to the classification of 771 lncRNAs based on their predicted functions, we further classified these lncRNAs according to their genomic locations. Six genomic location-based categories of lncRNAs were identified: intergenic (325 out of 771 lncRNAs, 42.15%), antisense (305 [39.55%]), divergent (74 [9.60%]), convergent (96 [12.45%]), divergent & convergent (31[4.02%]), and intronic (3 [0.39%]) (**Figure 1C**). We then characterized the features of lncRNAs, such as average length, exon number, isoform number, and expression level, in each category, and compared the results with the features of protein-coding transcripts. The lncRNAs were shorter (average length = 878.757 bp) and contained fewer exons (average exon number = 1.72) than coding transcripts (2215.58 bp and 4.6 exons, respectively) (*p* < 0.0001, Mann-Whitney *U*-test, two-tailed) (**Figures 1D, E**). On the other hand, the number of isoforms of lncRNAs (1.34) was comparable with that of coding RNAs (1.6) (**Figure 1F**). The median expression levels of lncRNAs (average TPM along leaf age) were significantly (11-fold) lower than those of coding transcripts (*p* < 0.0001, Mann-Whitney U-test, two-tailed) (**Figure 1G**). These results are consistent with those of previous studies, which identified lncRNAs involved in other biological processes (Di et al., 2014; Tsai et al., 2022). The RNA-seq read coverage of the novel lncRNAs identified in this study was confirmed using gene viewer (**Supplementary Figure 1A**), and their expression levels were validated through the RT-PCR analysis of eight randomly-selected transcripts (**Supplementary Figures 1B, C**).

### Identification of AR-lncRNAs

**Figure 2:**
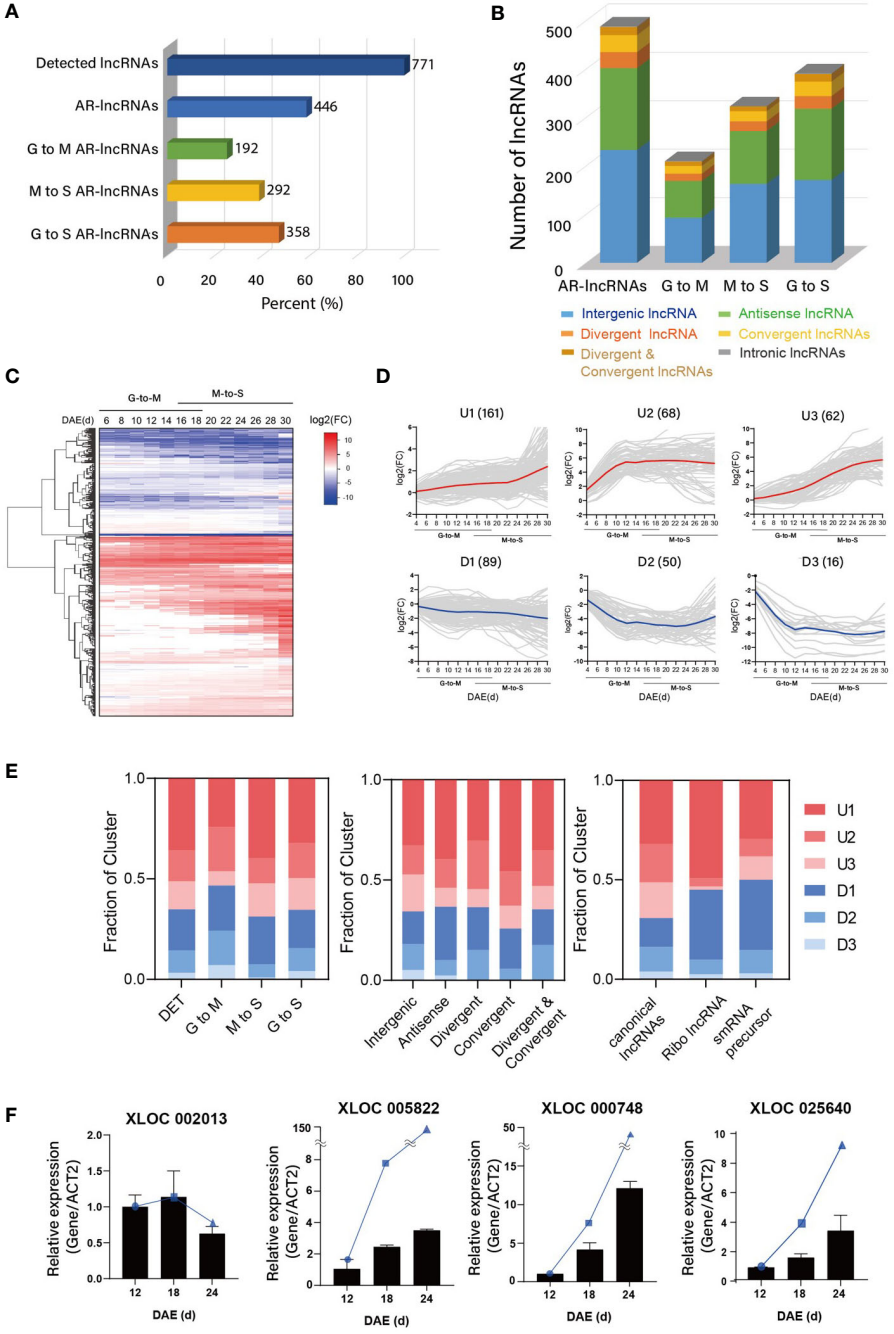
Identification of age-related lncRNAs (AR-lncRNAs) in *Arabidopsis*. **(A)** Proportions of lncRNAs detected in the reconstructed leaf transcriptome and differentially expressed in aging leaves (fold change ≥ 2, *p* <= 0.05). The leaf lifespan was divided into two developmental stages, G-to-M (Growth to Maturation, 4–18 d) and M-to-S (Maturation to Senescence, 16–30 d). Some of G-to S AR-lncRNAs are overlapped with G-to-M AR-lncRNAs or M-to-S AR-lncRNAs. **(B)** Proportions of AR-lncRNAs at different genomic locations with respect to the nearest protein-coding gene. **(C)** Heat maps showing the expression of AR-lncRNAs over the entire leaf lifespan. Rows are ordered based on hierarchical clustering. Color bar represents the gradient of log2(fold change) values relative to the 4-DAE time point. DAE: days after emergence. **(D)** Changes in AR-lncRNA transcript levels in aging leaves, as shown by k-means clustering. Six major clusters (upregulated, U1–U3; downregulated, D1–D3) were detected, depending on AR-lncRNA expression patterns. **(E)** Proportions of major AR-lncRNAs in different categories established based on the different developmental stages of leaves and the genomic locations and functions of AR-lncRNAs. **(F)** Expression analysis of novel AR-lncRNAs at three timepoints by qRT-PCR. Error bars represent the standard error of mean (SEM; *n* = 3). The blue circles, squares, and triangles means the TPM value of each lncRNA at indicated leaf ages (12d, 18d, 24d) from RNA-seq.

The functional transition of leaves during aging inspired us to examine the dynamic landscapes of lncRNAs. Of the 771 lncRNAs identified in this study, 446 (57.8%) were differentially expressed during leaf aging, as examined by DEseq2 (Love et al., 2014) (|log2(fold change)| ≥ 1, adjusted *p*-value [*p_adj_*] ≤ 0.05) (**Figure 2A**). Among the AR-lncRNAs, 192 and 292 lncRNAs showed dynamic changes of their expressions during the early biogenesis period (from growth [G] to maturation [M], 4–18 d) and late degeneration period (from M to senescence [S], 16–30 d), respectively, indicating that lncRNAs play important roles in leaf development. The majority of AR-lncRNAs were intergenic (44.39%), followed by antisense (37.44%), divergent (9.86%), convergent (12.11%), divergent & convergent (4.04%), and intronic (0.22%). The proportions of genomic location of AR-lncRNAs were similar to that of detected lncRNAs, and not significantly different between the G→M and M→S transitions (**Figure 2B**). Notably, the number of senescence-associated AR-lncRNAs (M→S) was greater than that of biogenesis associated-lncRNAs (G→M), suggesting that lncRNAs are more relevant to the leaf senescence process than to the leaf biogenesis process.

Differentially expressed lncRNAs were categorized into six major clusters, including three upregulated (U1–U3) and three downregulated (D1–D3) clusters, based on their expression kinetics over time, which represents 97% of the AR-lncRNAs (**Figures 2C, D**). We also examined the temporal expression profiles of lncRNAs showing age-dependent changes in transcript levels during the G→M and M→S transitions. During the G→M transition, the numbers of upregulated and downregulated AR-lncRNAs were similar (54% and 46%, respectively) (**Figure 2E**). Intriguingly, the majority of AR-lncRNAs (69%) were upregulated during the M→S transition. AR-lncRNA were preferentially upregulated, regardless of their genomic location (**Figure 2E**). Canonical lncRNAs were also preferentially upregulated, but rather similar numbers of ribo-lncRNAs and sRNA-precursor lncRNAs were up- and downregulated. The expression patterns of four randomly-selected AR-lncRNAs were verified by RT-PCR (**Figure 2F**).

Studies show that lncRNAs localize to various subcellular organelles, and regulate gene expression at various levels (transcriptional, post-transcriptional, and translational) (Bridges et al., 2021). Therefore, knowledge of the subcellular localization patterns of lncRNAs would provide information for inferring their gene regulation mode. We determined the subcellular localization of each AR-lncRNA by analyzing the publicly available transcriptome data of the cytosolic and nuclear fractions of 2-week-old *Arabidopsis* seedlings (Zhao et al., 2018). Of the 287 AR-lncRNAs identified in these transcriptomes, 211 (73.52%) were predominantly present in the nuclear fraction, whereas only 76 (26.48%) were enriched in the cytosolic fraction (**Supplementary Figures 2A, B** and **Supplementary Data 5**). This pattern was robust, regardless of the functional categories of AR-lncRNAs. For instance, both sRNA-precursor lncRNAs (19/23 [81.61%]) and sORF-encoding lncRNAs (56/93 [60.22%]) were more abundantly localized in the nuclear fraction. To confirm this result, we performed qRT-PCR on six randomly-selected lncRNAs using cDNA isolated from the nuclear and cytosolic fractions of 2-week-old seedlings, which validated the subcellular localization of all, but one, lncRNAs (**Supplementary Figure 2C**).

Leaf development is an integrated response of plants to an innate developmental program and environmental stress responses. Thus, some of the genes involved in leaf senescence are expected to control environmental responses. We therefore investigated whether AR-lncRNAs are regulated by stress responses using the previously published transcriptome data of ABA-, drought-, and cold-treated *Arabidopsis* (Zhao et al., 2018). Among the lncRNAs differentially expressed by the ABA, drought, or cold treatment, a large proportion (68/102 [66.7%], 72/112 [64.3%], and 70/127 [55%], respectively) was also affected by the developmental age, suggesting an extensive overlap between leaf senescence and stress responses. This result is consistent with that of the previous study on protein coding mRNAs (Zhao et al., 2018) (**Supplementary Figures 3A, B** and **Supplementary Data 4**).

### Identification of antisense lncRNAs overlapped with neighboring protein-coding genes and functional validation of putative lncRNA in the regulation of leaf senescence

**Figure 3:**
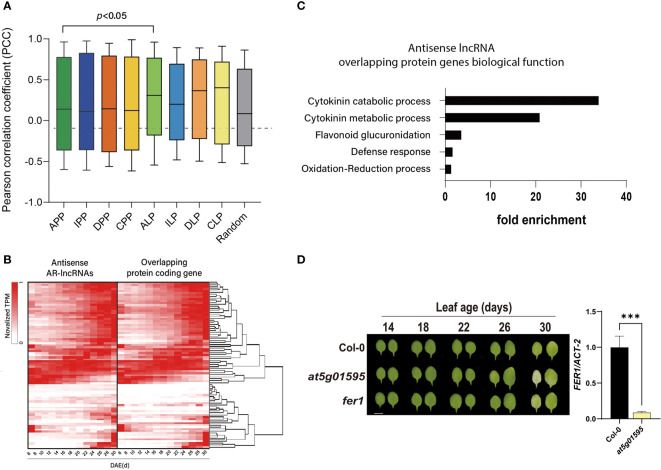
Expression correlation of antisense AR-lncRNAs and overlapping protein-coding genes during the leaf lifespan. **(A)** Box-plot displaying the expression correlation between AR-lncRNAs and adjacent genes. PCC: Pearson correlation coefficient; APP: overlapping protein-coding gene pairs; IPP: protein-coding genes and non-overlapping protein-coding gene pairs; DPP: divergent protein-coding gene pairs; CPP: convergent protein-coding gene pairs; ALP: antisense AR-lncRNAs and overlapping protein-coding genes; ILP: intergenic AR-lncRNAs and non-overlapping adjacent genes; DLP: divergent AR-lncRNAs and adjacent genes with a common promoter region; CLP: convergent AR-lncRNAs and adjacent genes within 1,000 bp; Random: random pairs. Central lines represent the mean. Whiskers represent the maximum and minimum values. Statistical difference is indicated by p-value (Mann- Whitney U-test. **(B)** Heat maps representing the expression pattern of antisense AR-lncRNAs and overlapping protein-coding genes. Rows are ordered based on hierarchical clustering. Columns indicate the number of days after emergence (DAE). Color bar shows the fraction density during the leaf lifespan. **(C)** Gene ontology biological process [GOBP of protein-coding genes overlapped with antisense AR-lncRNAs (p < 0.05)]. Fold enrichment represents the ratio of the proportion of input genes involved in GOBP and the proportion of genes in the given GOBP in involved in the background. **(D)** Phenotype of Col-0, *at5g01595* and *fer1* during developmental leaf senescence (left) and expression level of *FER1* mRNA was measured by qRT-PCR (right). Data in the right panel represent the mean of 3 biological replicates, and error bars represent SD (n = 3). Asterisks indicate significant differences (t -test; ***, p < 0.001).

Expression levels of some lncRNAs are significantly correlated with those of their neighboring protein-coding genes. Several lncRNAs are also known to control the expression of nearby genes (Statello et al., 2021), suggesting that these lncRNAs act as *cis*-regulators of genes. Thus, the positional relationship between lncRNAs and mRNAs in the genome would be important for predicting the lncRNA-controlled regulation of nearby genes. To infer the effect of lncRNAs on the expression of neighboring genes in aging leaves, we estimated the degree of co-expression between AR-lncRNAs and their adjacent protein-coding genes by calculating the Pearson correlation coefficients (PCCs). The co-expression of all pairs of age-related protein-coding genes was also analyzed for comparison. Notably, the PCC between pairs of antisense AR-lncRNAs and overlapping protein-coding genes was significantly higher than that between overlapping protein-coding gene pairs (*p* < 0.05, Mann-Whitney U-test, two-tailed) (**Figure 3A**). This result implies that antisense AR-lncRNAs act as *cis*-regulators of adjacent genes during leaf aging. Of the 168 antisense AR-lncRNAs, the TPM fractional density of 72 antisense AR-lncRNAs and their overlapping protein-coding genes pairs (PCC > 0.7) was visualized as a heatmap (**Figure 3B**). Gene ontology biological process (GOBP) enrichment analysis revealed that the neighboring protein-coding genes of antisense lncRNAs were significantly enriched by cytokinin catabolic/metabolic process, flavonoid glucuronidation, defense response, and oxidation-reduction processes (**Figure 3C**). Cytokinin is a representative hormone that negatively regulates leaf senescence in plants. The expression of cytokinin biosynthetic genes decreases, while that of cytokinin degradation genes increases during leaf senescence in *Arabidopsis* (Buchanan-wollaston et al., 2003; Breeze et al., 2011; Statello et al., 2021). Genes encoding two *SOB five-like* (*SOFL*) genes, *AtSOFL1* and *AtSOFL2*, which act as positive regulator of cytokinin levels and cytokinin-mediated development including longevity (Zhang et al., 2009), are found to be overlapped with *AT1G26210* and *AT1G26208* AR-lncRNAs, respectively. Antisense AR-lncRNA *AT3G63445* is overlapped with *CYTOKININ OXIDASE* (*CKX*) *6* that catalyzes the degradation of cytokinin.

Defense responses are also one of the typical age-associated biological processes (Kus et al., 2002; Mao et al., 2017). *FERRITIN1* (*FER1*), overlapped with antisense AR-lncRNA *AT5G01595*, plays a role in iron hemostasis and accumulates upon exposure to oxidative stress or to pathogen attack, as well as developmental factor. Mutation of *FER1* causes earlier onset of leaf senescence (Murgia et al., 2007). So, it is likely that *AT5G01595* lncRNA might play a role in leaf senescence possibly through modulating *FER1*. To validate the functional role of *AT5G01595*, the senescence phenotype of the third and fourth leaves of knockout line (*at5g01595*) and wild-type (Col-0) plants during age-dependent natural senescence was compared (**Figure 3D**). Initiation of leaf yellowing, which is an indicator of chloroplast senescence in mesophyll cells, occurred earlier in *at5g01595* than in Col-0. The photochemical efficiency (F_v_/F_m_), a representative physiological marker of leaf senescence, also declined rapidly. The early leaf senescence phenotype of *at5g01595* was further confirmed by analyzing the expression of the *SENESCENCE-ASSOCIATED GENE 12* (*SAG12*) and *CHLOROPHYLL A/B-BINDING PROTEIN 2* (*CAB2*), the molecular markers of leaf senescence (**Supplementary Figures 4B, C**). We then tested the effect of *AT5G01595* antisense lncRNA on the expression of *FER1*. The result showed that *FER1* transcript level was significantly lower in *at5g01595* than in Col-0 leaves (**Figure 3D**). These results indicate that *AT5G01595* potentially contributes to leaf senescence by modulating *FER1*.

Together, these findings suggest that leaves might utilize antisense lncRNAs as a regulatory program for controlling biological processes, particularly cytokinin-related processes and defense responses, throughout its lifespan.

### Identification of the potential regulatory network involving competitive endogenous AR-lncRNAs.

**Figure 4:**
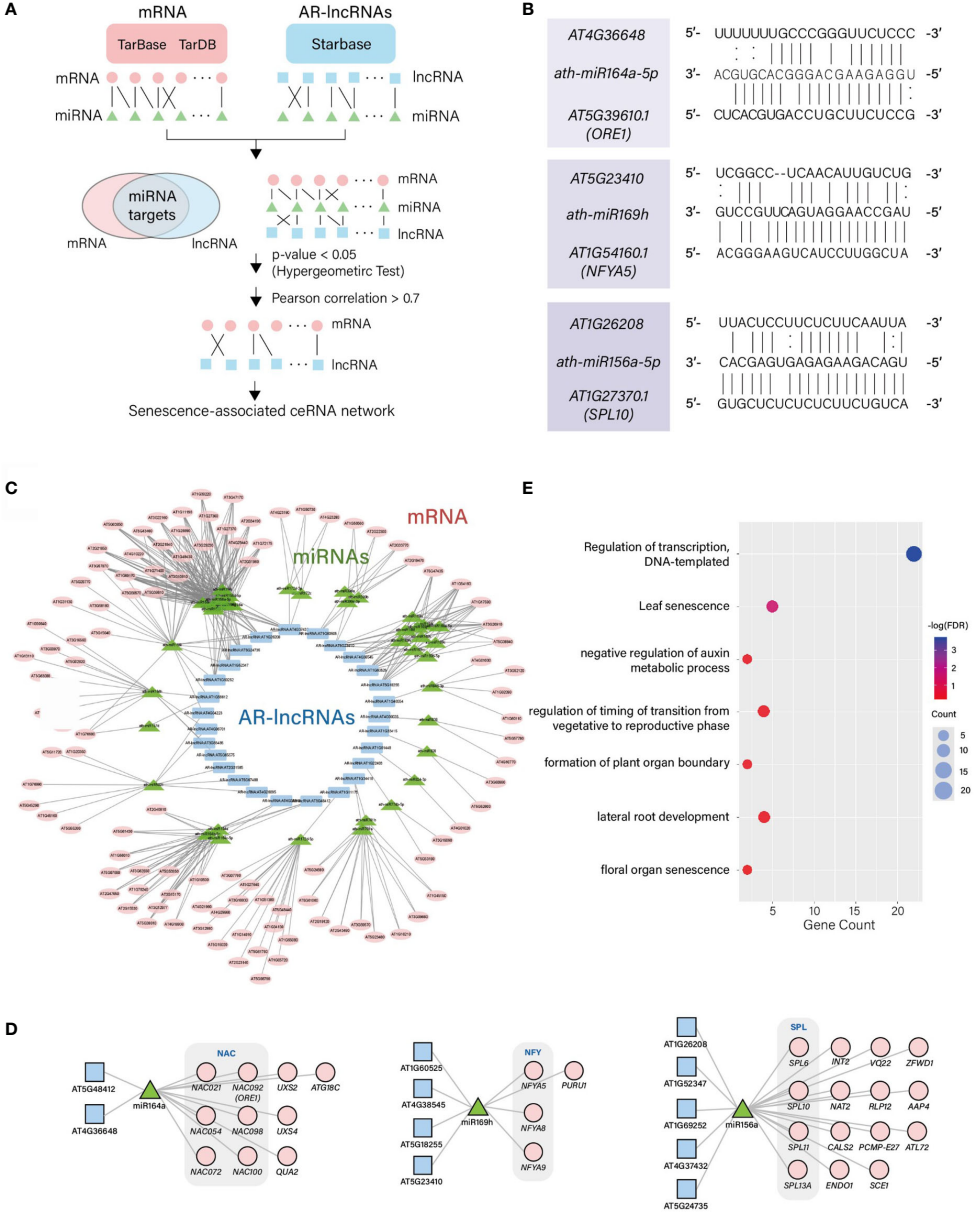
Identification of ceRNA sets containing AR-lncRNAs involved in leaf senescence. **(A)** Framework used to predict the ceRNA sets containing AR-lncRNAs. **(B)** Alignment of representative miRNAs and AR-lncRNAs. **(C)** ceRNA regulatory networks. **(D)** Three representative network modules showing the identified leaf senescence-related ceRNA sets. **(E)** Enriched GOBPs of mRNAs involved in ceRNA sets.

The lncRNAs can regulate mRNAs by sequestering specific miRNAs and mimicking their target recognition sequence in organisms. We searched for potential competitive endogenous RNAs (ceRNAs) involved in lncRNA–miRNA–mRNA interactions. We first integrated the TarDB (Liu et al., 2021) and TarBase (Karagkouni et al., 2018) datasets to search for mRNA–miRNA interactions, and StarBase (Li et al., 2014) to search for lncRNA–miRNA interactions in *Arabidopsis*. Using the hypergeometric test, potential ceRNA sets (lncRNA–miRNA–mRNA) were identified (**Figure 4A**) by evaluating the significance of interacting miRNAs shared by both mRNAs and lncRNAs (*p* < 0.05); these shared miRNAs were used as a junction. Among the potential ceRNA sets, we further narrowed down high-confidence ceRNA sets by calculating the PCCs of lncRNAs and their cognate mRNAs. The selection of lncRNAs and mRNAs with PCC > 0.7 led to the identification of 602 positively-correlated ceRNA sets (**Figure 4A** and **Supplementary Data 6**). Eleven of the identified lncRNAs that paired with ceRNAs would likely compete with miRNAs involved in leaf development or leaf senescence, such as miR156, miR164, and miR169 (**Figure 4B**). Among these miRNAs, miRNA164, a negative regulator of leaf senescence, is known to mediate the cleavage of *ORESARA1* (*ORE1*), which induces cell death and leaf senescence (Kim et al., 2009). One of the AR-lncRNAs, *AT4G36648*, was identified as a target of *miRNA164* in this analysis, which involves in the ceRNA set linking *AT4G36648-miRNA164-ORE1*. Both *AT4G36648* and *ORE1* showed a rapid change in expression at the late degeneration stage. A strong positive correlation between *AT4G36648* and *ORE1* (PCC = 0.99) supports the presence of ceRNA that anchors these transcripts through *miRNA164*. Moreover, *AT4G36648* was expressed during the M→S transition, which suggests the regulatory role of this ceRNA in leaf senescence. We also identified *AT5G23410-miR169-NUCLEAR FACTOR Y* (*NF-Y*) as ceRNA set. The expression level of the AR-lncRNA *AT5G23410*, followed by that of *miR169*-targeted *NFYA5*, which is known to modulate ABA-dependent stress responses (Li et al., 2008), was induced during aging. In this ceRNA set, the expression level of *AT5G23410* was highly correlated with that of *NFYA5* (PCC = 0.90). ABA is one of the hormones accelerating leaf senescence. Thus, this module is likely to be involved in mediating crosstalk between leaf senescence and stress responses. The AR-lncRNA *AT1G26208* was identified as an interacting partner of *miRNA156*, which inhibits the action of *SQUAMOSA PROMOTER BINDING-LIKE* (*SPL*). Both *miRNA156* and *SPL* form a regulatory module to control age-dependent developmental transition as well as abiotic and biotic stress responses (Wang et al., 2009; Ma et al., 2021b). Similar to the above two ceRNA sets, the age-dependent expression levels of AR-lncRNA *AT1G26208* and *SPL10* were highly correlated (PCC = 0.98), implying that the AR-lncRNA *AT1G26208* modulates age-dependent pathways or integrates aging cues with stress responses.

Next, we reconstructed the ceRNA (lncRNA–miRNA–mRNA) interaction network. This network is composed of 106 mRNAs differentially expressed during aging, and 27 AR-lncRNAs which are commonly targeted by 38 miRNAs (**Figures 4C, D** and **Supplementary Data 6**). To predict the potential function of AR-lncRNAs comprising the ceRNA network, the interacting mRNAs in the ceRNA network were subjected to the GOBP enrichment analysis. These genes were over-represented by the regulation of transcription and leaf senescence, suggesting that AR-lncRNAs perform an important regulatory role during leaf aging by participating in the ceRNA network (**Figure 4E**).

### Identification of potential AR-lncRNAs interacting with mRNAs and functional validation of two lncRNAs in the regulation of leaf senescence

**Figure 5:**
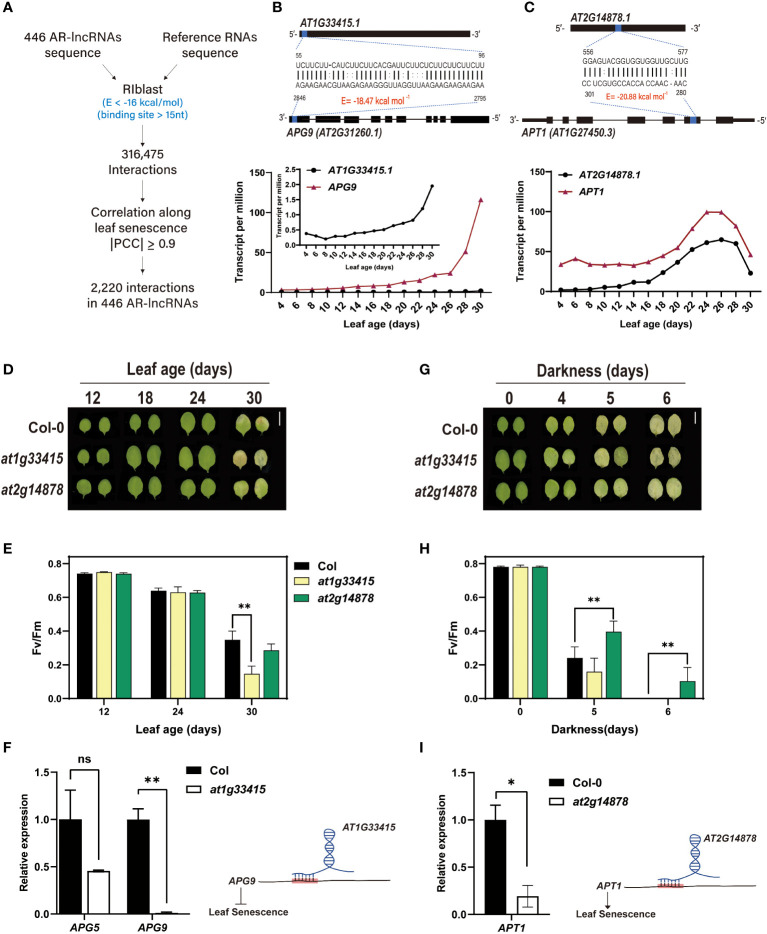
Prediction and validation of leaf senescence-regulating AR-lncRNAs. **(A)** Computational pipeline for the prediction of putative AR-lncRNA-RNA interactions. Interaction energy (**E**, kcal/mol) of AR-lncRNA-RNA pairs was calculated using RIBLAST 1.1.1. Expression correlation along leaf aging was used to reduce the number of AR-lncRNA-RNA pairs, resulting in the identification of 2,220 candidate pairs (|PCC| ≥ 0.9). **(B, C)** Interaction between *AT1G33415* and *APG9* **(B)** and between *AT2G14878* and *APT1* **(C)** (upper panel), and age-dependent expression patterns of *AT1G33415* and *APG9* **(B)** and *AT2G14878* and *APT1* **(C)** (lower panel). Expression levels were expressed as the mean TPM values of two biological replicates. **(D, E)** Leaf yellowing phenotype **(D)** and F_v_/F_m_ ratio **(E)** of the third and fourth rosette leaves of *at1g33415* and *at2g14878* mutants during natural leaf senescence. Error bars represent standard deviation (SD; *n* = 4). Asterisks indicate significant differences between the indicated groups (t- test; **, p < 0.01). **(G, H) (G)** Leaf yellowing phenotype **(G)** and **(H)** F_v_/F_m_ ratio **(H)** of *at1g33415* and *at2g14878* mutants subjected to dark-induced leaf senescence. Error bars represent SD (*n* = 6), and asterisks indicate significant differences between the indicated groups (t-test; **, p < 0.01). **(F)** Expression analysis of *APG5* and *APG9* in wild-type (Col-0) and *at1g33415* leaves by qRT-PCR (left), and *AT1G33415*-mediated suppression of leaf senescence potentially through interaction with *APG9* mRNA (right). **(I)** Expression analysis of *APT1* in wild-type (Col-0) and *at2g14878* leaves by qRT-PCR (left), and *AT2G14878*-mediated acceleration of leaf senescence potentially through the stabilization of *APT1* mRNA (right). In **(F)** and **(I)**, data in the left panel represent the mean of two replicates, and error bars represent SD (*n* = 2). Asterisks indicate significant differences (t-test; ns, non-significance; *, p < 0.05; **, p < 0.01).

Numerous lncRNAs regulate gene expression by directly interacting with target mRNAs and affecting their stability or processing (Bardou et al., 2014; Sebastian-delaCruz et al., 2021). To identify putative mRNA-lncRNA pairs, we utilized RIBLAST, a computational tool used to predict comprehensive lncRNA–RNA interactions based on the seed-and-extension approach, where a target prediction was experimentally validated (Kretz et al., 2013; Fukunaga and Hamada, 2017b). We first calculated the interaction energy between the AR-lncRNA and mRNA sequences in the TAIR10 database, and then selected RNA segment pairs with < -16 kcal/mol interaction energy in 15 ≥ nt. This analysis led to the identification of 316,475 AR-lncRNA-mRNA pairs. These interacting pairs were further narrowed down based on the PCC values, resulting in the identification of highly co-expressed AR-lncRNA and target mRNA pairs (|PCC| ≥ 0.9) during the leaf lifespan. Through this analysis, we obtained 2,220 putative interactions among 446 AR-lncRNAs (**Figure 5A** and **Supplementary Data 7**). To explore AR-lncRNAs associated with leaf senescence, we searched for pairs of AR-lncRNAs and mRNAs whose genes are known to be involved in the regulation of leaf senescence (**Supplementary Data 8**) (Li et al., 2020). The predicted regulatory modules were further validated through the characterization of loss-of-function mutants. In this study, we focused on two AR-lncRNAs: *AT1G33415* and *AT2G14878* (**Figures 5B, C**).

Autophagy is required for nutrient recycling during leaf senescence. In *Arabidopsis*, Autophagy9 (APG9) is known to regulate the formation of autophagosomes, which are crucial for autophagy process, from the endoplasmic reticulum (Zhuang et al., 2017). The expression of *APG9* is markedly upregulated during leaf senescence, and the *apg9* mutant exhibits precocious senescence phenotypes, indicating that APG9 plays a negative role in leaf senescence (Zhuang et al., 2017). Given that *AT1G33415* interacts with *APG9* and the corresponding genes are highly co-expressed (**Figure 5B**), we decided to evaluate the involvement of *AT1G33415* in the regulation of leaf senescence.

Firstly, we compared the yellowing phenotype of the third and fourth leaves of mutant (*at1g33415*) and wild-type (Col-0) plants during age-dependent natural senescence. The progression of leaf yellowing occurred more rapidly in the mutant than in the wild type (**Figure 5D**). The early leaf senescence phenotype of *at1g33415* was confirmed by measuring the photochemical efficiency (F_v_/F_m_), which declined rapidly in *at1g33415* (**Figure 5E**). We also conducted a dark-induced leaf senescence assay using detached leaves. Similar to the leaf phenotype observed during age-dependent senescence, dark-induced senescence symptoms (leaf yellowing and F_v_/F_m_ decline) were also accelerated in the leaves of *at1g33415* (**Figures 5G, H**). *SAG12* expression was also analyzed for confirming the senescence phenotype (**Supplementary Figures 5A, B**). We then examined the expression level of *APG9* in mature Col-0 and *at1g33415* leaves. The results showed that *APG9* transcript levels were significantly lower in *at1g33415* than in Col-0 leaves (**Figure 5F**). These results imply that *AT1G33415* potentially contributes to the stabilization of *APG9* mRNA level *in trans* through RNA–RNA interaction, thereby playing a role as a negative regulator of leaf senescence.

*AT2G14878-ADENINE PHOSPHORIBOSYL TRANSFERASE 1* (*APT1*) was predicted as a component of another regulatory module. APT1 inactivates cytokinin by catalyzing its conversion from free bases to nucleotides, and the loss-of-function *apt1* mutant exhibits delayed leaf senescence in darkness (Zhang et al., 2013). The results of interaction energy analysis and the co-expression pattern of *APT1* and *AT2G14878* raised the possibility that *AT2G14878* regulates dark-induced leaf senescence involving cytokinin metabolism (**Figure 5C**). The *at2g14878* knockout mutant did not exhibit altered leaf senescence phenotype during aging (**Figures 5D, E**). However, similar to the extended longevity of *atp1* leaves under dark-induced leaf senescence, the *at2g14878* leaves showed a delayed senescence phenotype when leaf yellowing symptom was monitored in darkness (**Figure 5G**). The F_v_/F_m_ of *at2g14878* leaves was also maintained for a longer duration during dark incubation, supporting the positive role of *AT2G14878* in dark-induced leaf senescence (**Figure 5H**). The senescence phenotype was further confirmed by examining expression of *SAG12* (**Supplementary Figures 5A, B**). To determine whether delayed senescence in the *at2g14878* mutant is caused by the suppression of *APT1* expression, the transcript level of *APT1* was analyzed in Col-0 and *at2g14878* leaves. To rule out the possibility that *APT1* expression was altered by delayed senescence, leaves at the early maturation stage were utilized for this experiment. As expected, the transcript level of *APT1* was significantly suppressed in the mutant (**Figure 5I**). This suggests that *AT2G14878* might promote cytokinin metabolism during leaf senescence by interacting with *APT1*, which decreases the cytokinin content of aged leaves.

Overall, the AR-lncRNA-mRNA pairs identified in this study provide valuable information for elucidating the biological function of lncRNAs, and will help to explore new lncRNA-mediated regulatory pathways involved in leaf senescence.

## Discussion

Leaf development involves a series of functional and regulatory transitions from biogenesis to degeneration, which should be tightly regulated by coordinated molecular processes. Previously, we constructed a high-resolution and multidimensional transcriptome map to understand the fundamental transcriptional programs underlying age-dependent developmental shifts that occur during leaf development in *Arabidopsis*. Using these datasets, we performed comprehensive profiling of molecular processes active during leaf aging, and revealed coordinated transcriptional programs including transcriptional regulation by transcription factors and post-transcriptional regulation by various types of sRNAs (Woo et al., 2016).

Emerging evidence shows that lncRNAs play crucial roles in many biological processes and function through diverse mechanisms at multiple regulatory levels. However, the role of lncRNAs in leaf senescence regulation has not been investigated to date. Recently, a study in flag leaf senescence of rice reported the list of lncRNAs expressed during aging (Huang et al., 2021). Also, the study in the tomato analyzed lncRNA expression during the leaf senescence process (Li et al., 2022). However, the role of lncRNAs in *Arabidopsis* leaf senescence regulation has not been comprehensively investigated to date. In this study, we systematically identified 771 lncRNAs, including 539 annotated and 232 novel lncRNAs, during *Arabidopsis* leaf development. One of the challenges of lncRNA research in *Arabidopsis* is to explore the uncharacterized functions of lncRNAs. In general, bioinformatics tools predict certain transcripts as lncRNAs based on their sequence characteristics; however, these strategies are not suitable for inferring the function of lncRNAs. To overcome this problem, we employed several computational tools to infer the hidden functions of lncRNAs in leaf development and aging.

We integrated orthogonal sequencing datasets, such as ribo-seq data, to classify the 771 detected lncRNAs into three functional groups: sRNA precursors, canonical lncRNAs, and ribo-lncRNAs. In the ribo-lncRNA category, we identified novel lncRNAs that could potentially be translated into sORFs (sORF-encoding lncRNAs); however, experimental verification should be needed to determine whether these lncRNAs generate peptides/proteins as predicted, and under which conditions these peptides/proteins are actively expressed. To ascertain the potential functional contribution of small peptides to leaf development including senescence, it is necessary to examine the effect of mutations in the stop or start codons of the predicted small peptides. Some lncRNAs have also been reported to play dual roles; lncRNAs such as ENOD40 encode a small peptide as well as function as regulatory RNAs (Bardou et al., 2011). Other molecules embedded in lncRNAs are precursors of sRNAs such as miRNAs, which are processed from the introns of lncRNAs. These lncRNAs may not possess functions other than generating sRNAs; nonetheless, it is possible that processed lncRNAs act as modulators of target gene expression.

Of the 771 lncRNAs detected in leaves, 446 AR-lncRNAs were differentially expressed along aging. Intriguingly, the expression of AR-lncRNAs was regulated more dynamically in senescing leaves than in growing leaves (**Figure 2A**), revealing the contribution of these lncRNAs to leaf senescence. We also found that a large proportion of AR-lncRNAs (65.2%) was upregulated during leaf senescence (**Figure 2D**). Similarly, Huang et al. reported that the number of upregulated lncRNAs is higher than that of downregulated lncRNAs in late-senescence stage leaves compared to early booting stage leaves (FL1) in rice (Huang et al., 2021). Moreover, Li et al. observed a bigger number of upregulated genes than downregulated genes during tomato leaf senescence (Li et al., 2022). Such a characteristic expression pattern of AR-lncRNAs conserved in all three plant species might reflect that leaf senescence, despite its degenerative nature, involves a tightly-regulated program and has evolved to achieve biological processes that contribute to plants’ fitness, such as nutrient relocation.

Integration of AR-lncRNAs with other transcriptome datasets generated under stress conditions revealed that AR-lncRNAs strongly overlap with lncRNAs potentially involved in stress responses (**Supplementary Figure 3B**). Overlapping AR-lncRNAs might be involved in the protection of cellular integrity needed for the progression of leaf senescence, eventually leading to cell death. Our results, together with previous mRNA transcriptome data (Breeze et al., 2011; Woo et al., 2016), imply that leaf senescence is an intricate process, in which diverse environmental effects are superimposed on the age-dependent program. This mechanism would increase plant fitness in changing environments.

In this study, we used several different approaches to infer the biological function of AR-lncRNAs. Given that lncRNAs might regulate the expression of neighboring protein-coding genes *in cis* (Yang et al., 2013; Liu and Lim, 2018), the potential co-expression pattern of AR-lncRNAs and their cognate sense genes was first analyzed. Consistent with previous studies (Zhao et al., 2018), the expression of AR-lncRNAs with *cis*-NAT type was correlated with that of neighboring protein-coding genes. Notably, genes encoding proteins involved in cytokinin metabolic/catabolic processes were strongly enriched. Cytokinin is involved in cellular maintenance, suppressing senescence (Kim et al., 2006b). Thus, these AR-lncRNAs might be potential candidates that participate in leaf senescence by regulating cytokinin metabolism. We have also demonstrated that antisense AR-lncRNA *AT5G01595* might serve as a negative regulator in leaf senescence by modulating the expression of *FER1* that is an important player of iron-detoxification during leaf senescence.

ceRNAs play important roles in the regulation of biological processes; for example, IPS1 is involved in phosphate homeostasis. In the current study, we generated a list of developmental age-induced ceRNA networks in *Arabidopsis*, which will be useful to infer the physiological functions of AR-lncRNAs and their regulatory mode in the age-dependent program. *AT4G36648*-*miR164*-*ORE1* is a representative ceRNA network identified in this study that potentially regulates leaf senescence. ORE1 is one of the master transcriptional regulators of leaf senescence, and its expression must be elaborately regulated. *ORE1* is regulated at the transcriptional level by ETHYLENE INSENSITIVE 3 (EIN3), PHYTOCHROME-INTERACTING FACTOR 4 (PIF4), and PIF5 (Sakuraba et al., 2014), and at the post-transcriptional level by *miRNA164* (Kim et al., 2009). Our results suggest the AR-lncRNA *AT4G3664* as another regulator of *ORE1* expression, although we have not yet experimentally validated this finding. Functional analysis of *AT4G3664* would provide mechanistic insights into how a robust regulatory network involving *ORE1* is organized and how it functions to modulate leaf senescence.

Given that interactions with regulatory RNAs are important for coordinating gene expression and regulating mRNA stability or splicing as well as translation of target genes through base-pairing interactions, we calculated the RNA–RNA interaction potential and also utilized the co-expression analysis approach to infer the putative functions of AR-lncRNAs. The AR-lncRNA-mRNA pairs identified in this study may serve as an initial resource for exploring the hidden regulatory pathways of leaf senescence.

As a proof of concept, two AR-lncRNAs were tested using the genetic approach. Loss-of-function mutations of two AR-lncRNAs resulted in altered senescence symptoms, demonstrating that these two AR-lncRNAs are essential for modulating leaf senescence. Low levels of target mRNAs in AR-lncRNA mutants as well as highly correlated gene expression patterns of paired AR-lncRNAs and mRNAs further support that both AR-lncRNAs regulate the stability of target mRNAs. It should be noted that the expression of target genes was analyzed during the early leaf maturation phase to rule out the possibility that reduced expression of target gene in the mutants was caused by the altered leaf senescence phenotype. Targets of these lncRNAs were identified as genes involved in autophagy as well as cytokinin metabolism, and both these processes are important for leaf senescence. To fully elucidate the detailed regulatory mechanism of how AR-lncRNAs affect the stability of target transcripts, further experiments such as RNA-pulldown assays (which would reveal direct RNA–RNA interactions) and genetic analysis of transgenic plants with mutated interaction sites need to be conducted.

Overall comparison of lncRNAs identified in *Arabidopsis*, rice and tomato in the context of leaf senescence revealed interesting features. In the case of rice, in total 3953 lncRNA were identified, which is composed of intergenic non-coding RNAs (lincRNAs) (2262, 57.2%), antisense lncRNAs (1260, 31.9%), sense lncRNAs (338, 8.55%) and intronic lncRNAs (93, 2.35%). In the case of tomato, in total 2074 lncRNAs were identified including intergenic lncRNAs (~55%), intronic lncRNAs (~25%), bidirectional lncRNAs (~10%), sense lncRNAs (~5%), and antisense lncRNAs (~5%). If we compare our result in *Arabidopsis* with those other species, all three species show a similar proportion of intergenic lncRNAs, which is the largest category among others. However, it is interesting that the proportion of antisense lncRNAs in tomato (~5%) is much smaller than that of *Arabidopsis* (39.4%) and rice (31.9%). This suggests the existence of different usage of lncRNA categories participating in the regulation of leaf senescence in different plant species. Huang et al. also constructed ceRNA network linking mRNA-miRNA-lncRNA for flag leaf of rice, as we reported ceRNAs in this study for *Arabidopsis*. Interestingly, ceRNA modules involving *miR164* were detected in both Huang et al. (rice) and our study (*Arabidopsis*), which revealed the evolutionary conservation of the *miR164* functionality in leaf senescence *via* forming ceRNA network.

In terms of functionality, our study revealed that cytokinin catabolic process, flavonoid glucuronidation, defense response, and oxidation-reduction process is enriched in genes overlapped with antisense AR-lncRNAs during *Arabidopsis* leaf aging. Additionally, putative target genes of ceRNA network are involved in the processes such as regulation of transcription, leaf senescence, regulation of auxin metabolic process, formation of plant organ boundary, lateral root development, and floral organ senescence. Interestingly, enrichment of the oxidation-reduction process and regulation of transcription is commonly observed in the putative target genes of lncRNAs during rice leaf aging (Huang et al., 2021). Enrichment of oxidation-reduction related process in the putative target genes of lncRNAs is also conserved in tomato leaf senescence (Li et al., 2022). Huang et al. study additionally revealed that lipid metabolic process, transmembrane transport, and response to hormone processes are significantly enriched by the target genes of lncRNAs during rice leaf aging. In the case of leaf senescence in tomato, photosynthesis and starch and sucrose metabolism are additionally enriched by target genes of lncRNAs. These comparisons showed the existence of shared and distinct biological processes regulated by target genes of lncRNAs during leaf senescence in different plant species.

Based on the above-mentioned bioinformatics analyses, we not only systematically identified the lncRNAs over the leaf lifespan but also comprehensively reported the potential roles of these lncRNAs. Our predictions will open a new avenue for understanding *Arabidopsis* lncRNAs, by providing a comprehensive and confident list of lncRNA sets, and highly likely novel interactions between lncRNAs and mRNAs. Also in the future molecular studies, instead of focusing on one layer of molecules such as lncRNAs, mRNAs, or miRNAs individually, their network module needs to be experimentally investigated altogether, to study the emergent function of those networks which couldn’t be uncovered by experiments focusing on the individual species.

## Data availability statement

Publicly available datasets were reanalyzed in this study. The data used in the study are deposited in the Gene Expression Omnibus (GEO) repository, accession numbers are as follows: GSE42695, GSE120709, GSE43616, GSE69802.

## Author contributions

JK, JuhL, MK, HJ, and PL designed research. JK, JuhL, MK, and JusL analyzed data. HL and TT performed research. JK, JuhL, MK, HJ, and PL wrote the paper. All authors contributed to the article and approved the submitted version.

## Funding

This research was supported by the Mid-career Researcher Program (2019R1A2C1089459) and Basic Research Laboratory Program (2020R1A4A1019408) through the National Research Foundation of Korea (NRF) funded by the Ministry of Science (to P.O.L.)

## Conflict of interest

The authors declare that the research was conducted in the absence of any commercial or financial relationships that could be construed as a potential conflict of interest.

## Publisher’s note

All claims expressed in this article are solely those of the authors and do not necessarily represent those of their affiliated organizations, or those of the publisher, the editors and the reviewers. Any product that may be evaluated in this article, or claim that may be made by its manufacturer, is not guaranteed or endorsed by the publisher.

## Supplementary material

Supplementary Figure 1 Coverage and RT-PCR data of *Arabidopsis* lncRNAs. **(A)** Gene view of the mRNA coverage of novel lncRNAs. **(B, C)** Validation of novel lncRNAs by RT-PCR analysis of total RNA.

Supplementary Figure 2 AR-lncRNAs mainly localize to the nucleus. **(A)** Heat map showing the relative abundances of AR-lncRNAs in the cytosolic and nuclear fractions of RNA samples prepared from 2-week-old seedlings. Rows are ordered based on hierarchical clustering. Color bar shows the fraction density between cytosol and nucleus. **(B)** Summary of sub-localization of lncRNAs and AR-lncRNAs. **(C)** Expression analysis of representative novel AR-lncRNAs in the cytosolic and nuclear fractions of RNA samples by qRT-PCR. Color of the AR-lncRNA names indicate the categories based on their localization as shown in **(B)** (pink: Nucleus only, orange: Nucleus > Cytosol, dark blue: Cytosol only, skyblue: Cytosol > Nucleus).

Supplementary Figure 3 AR-lncRNAs are differentially expressed under stress conditions. **(A)** Heat maps representing the differentially expressed lncRNAs (DE-lncRNAs) in *Arabidopsis* plants treated with ABA, drought, and cold (*p* < 0.05, |log2(fold change) | ≥ 1). Rows are ordered based on hierarchical clustering. Color bar represents the gradient of log2(fold change) values compared with the before-treatment control. **(B)** Venn diagram indicating the numbers of lncRNAs showing differential expression in senescence, ABA, drought, and cold treatments.

Supplementary Figure 4 Genomic structure of *FER1* and *AT5G01595* and changes in senescence-associated gene expression during developmental leaf senescence. **(A)** Schematic structure of *FER1* and *AT5G01595*. The filled boxes for exons and the T-DNA insertion sites are indicated. **(B, C)** Senescence marker genes expression in wild-type (Col-0) and *at5g01595*. (B) *SAG12* and **(C)** *CAB2* expressions were detected by qRT-PCR in third and fourth leaves along the leaf age.

Supplementary Figure 5 Changes in senescence-associated gene expression in *at1g33415 and at2g14878* during developmental and dark-induced leaf senescence. **(A, B)** Expression analysis of *SAG12* in wild-type (Col-0), *at1g33415* and *at2g14878* leaves during developmental **(A)** and dark-induced leaf senescence (B) by qRT-PCR. In **(A)** and **(B)**, data the mean of two replicates, and error bars represent SD (*n* = 2). Statistical analysis was performed using student’s t-test (*, p < 0.05; **, p < 0.01; ***, p<0.001).

Supplementary Data Sheet 2 Annotated lncRNAs.
