# Supplementary figures and images for: Dynamic landscape of long noncoding RNAs during leaf aging in Arabidopsis
Source: Front Plant Sci. 2022 Dec 1;13:1068163. doi: 10.3389/fpls.2022.1068163 (PMC9753222; doi:10.3389/fpls.2022.1068163)

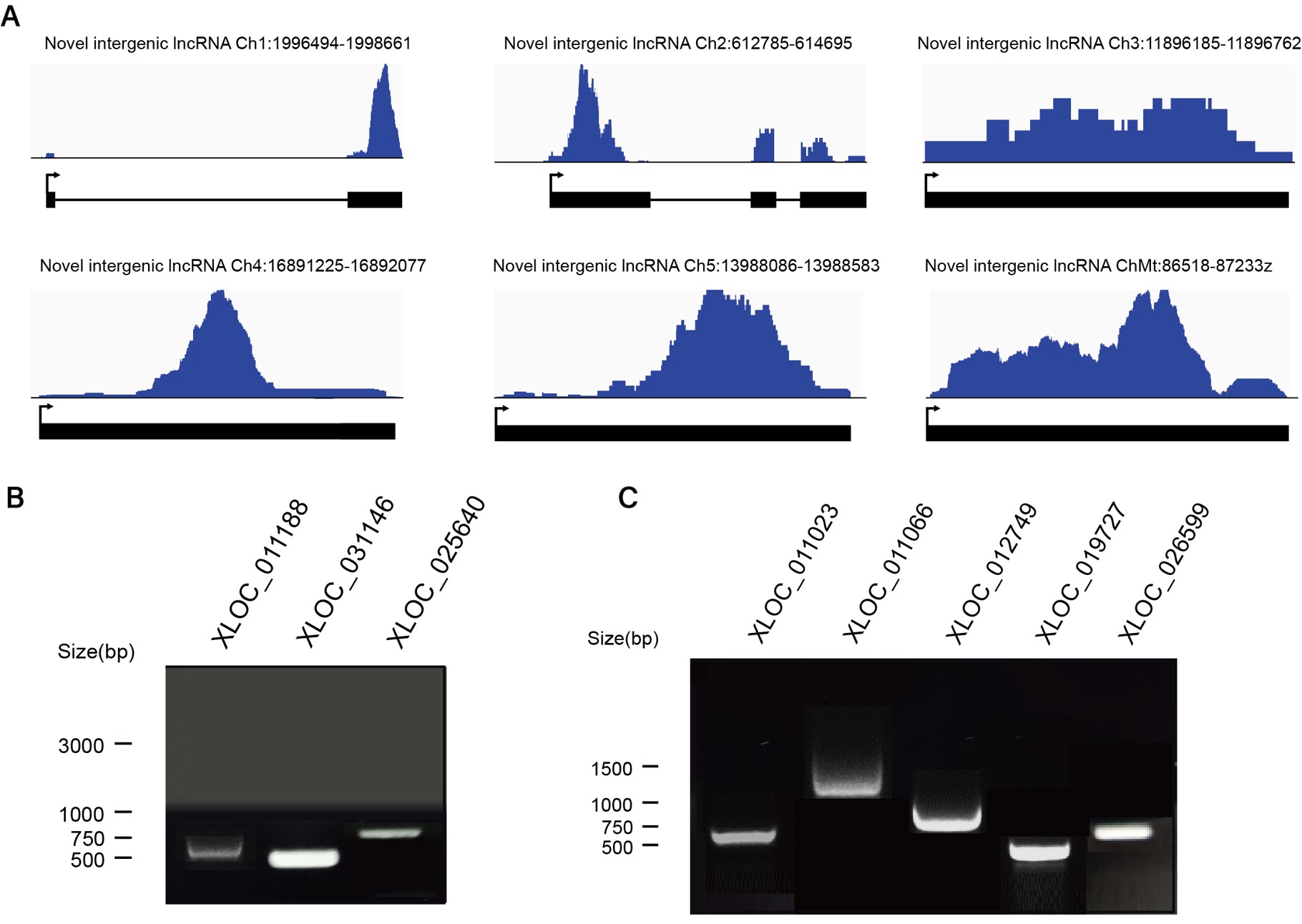

Supplement: Supplementary Figure 1 — Coverage and RT-PCR data of Arabidopsis lncRNAs. (A) Gene view of the mRNA coverage of novel lncRNAs. (B, C) Validation of novel lncRNAs by RT-PCR analysis of total RNA. [file Image_1.jpeg]

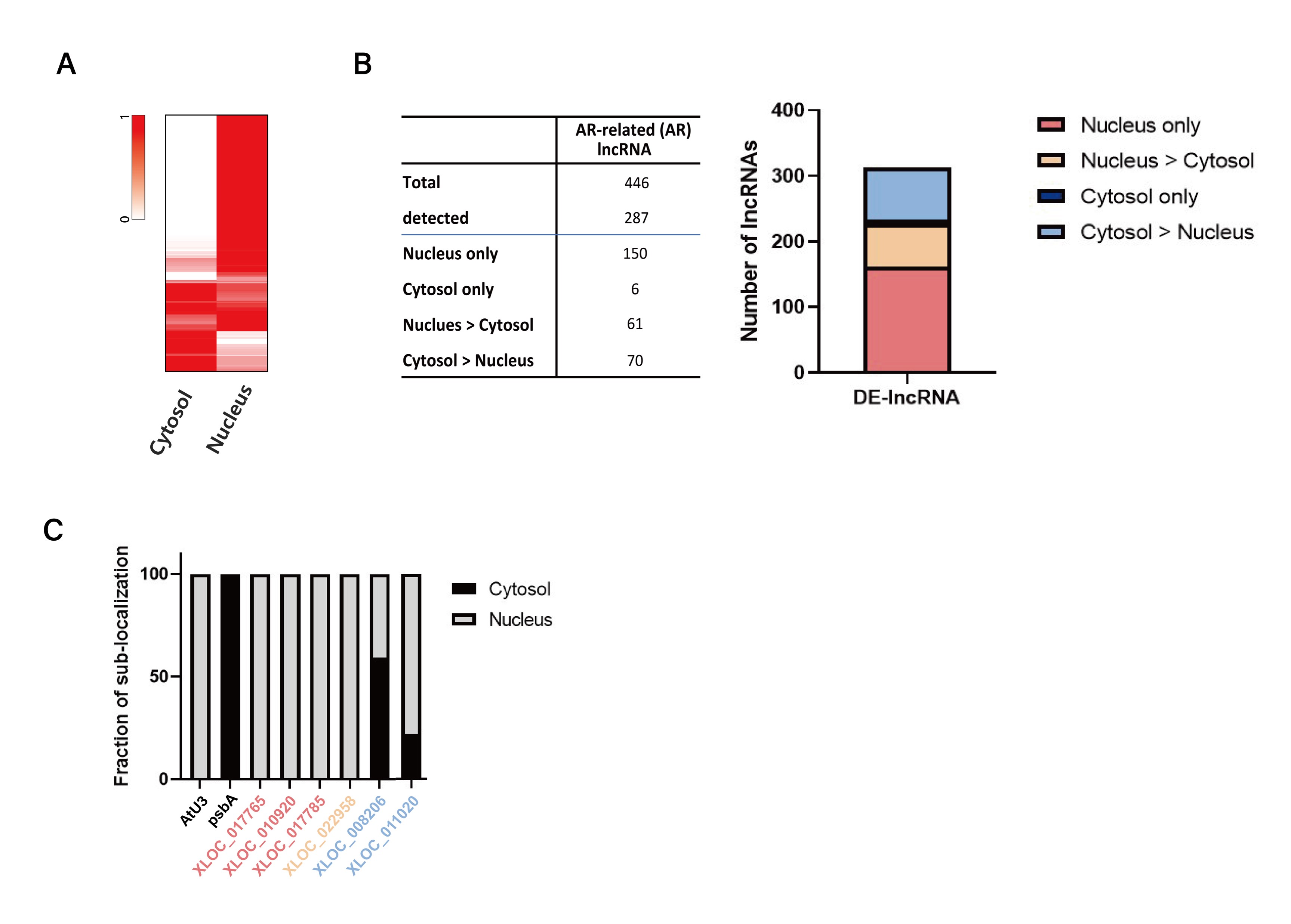

Supplement: Supplementary Figure 2 — AR-lncRNAs mainly localize to the nucleus. (A) Heat map showing the relative abundances of AR-lncRNAs in the cytosolic and nuclear fractions of RNA samples prepared from 2-week-old seedlings. Rows are ordered based on hierarchical clustering. Color bar shows the fraction density between cytosol and nucleus. (B) Summary of sub-localization of lncRNAs and AR-lncRNAs. (C) Expression analysis of representative novel AR-lncRNAs in the cytosolic and nuclear fractions of RNA samples by qRT-PCR. Color of the AR-lncRNA names indicate the categories based on their localization as shown in (B) (pink: Nucleus only, orange: Nucleus > Cytosol, dark blue: Cytosol only, skyblue: Cytosol > Nucleus). [file Image_2.jpeg]

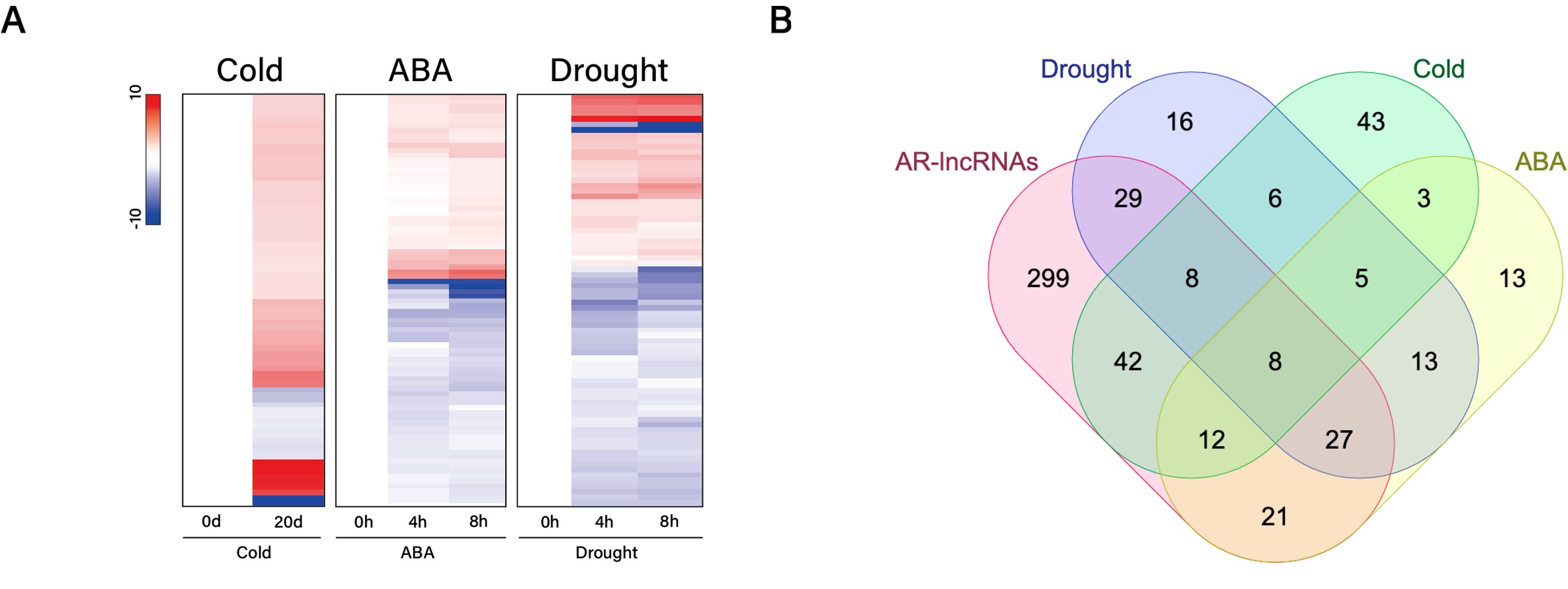

Supplement: Supplementary Figure 3 — AR-lncRNAs are differentially expressed under stress conditions. (A) Heat maps representing the differentially expressed lncRNAs (DE-lncRNAs) in Arabidopsis plants treated with ABA, drought, and cold (p < 0.05, |log2(fold change) | ≥ 1). Rows are ordered based on hierarchical clustering. Color bar represents the gradient of log2(fold change) values compared with the before-treatment control. (B) Venn diagram indicating the numbers of lncRNAs showing differential expression in senescence, ABA, drought, and cold treatments. [file Image_3.jpeg]

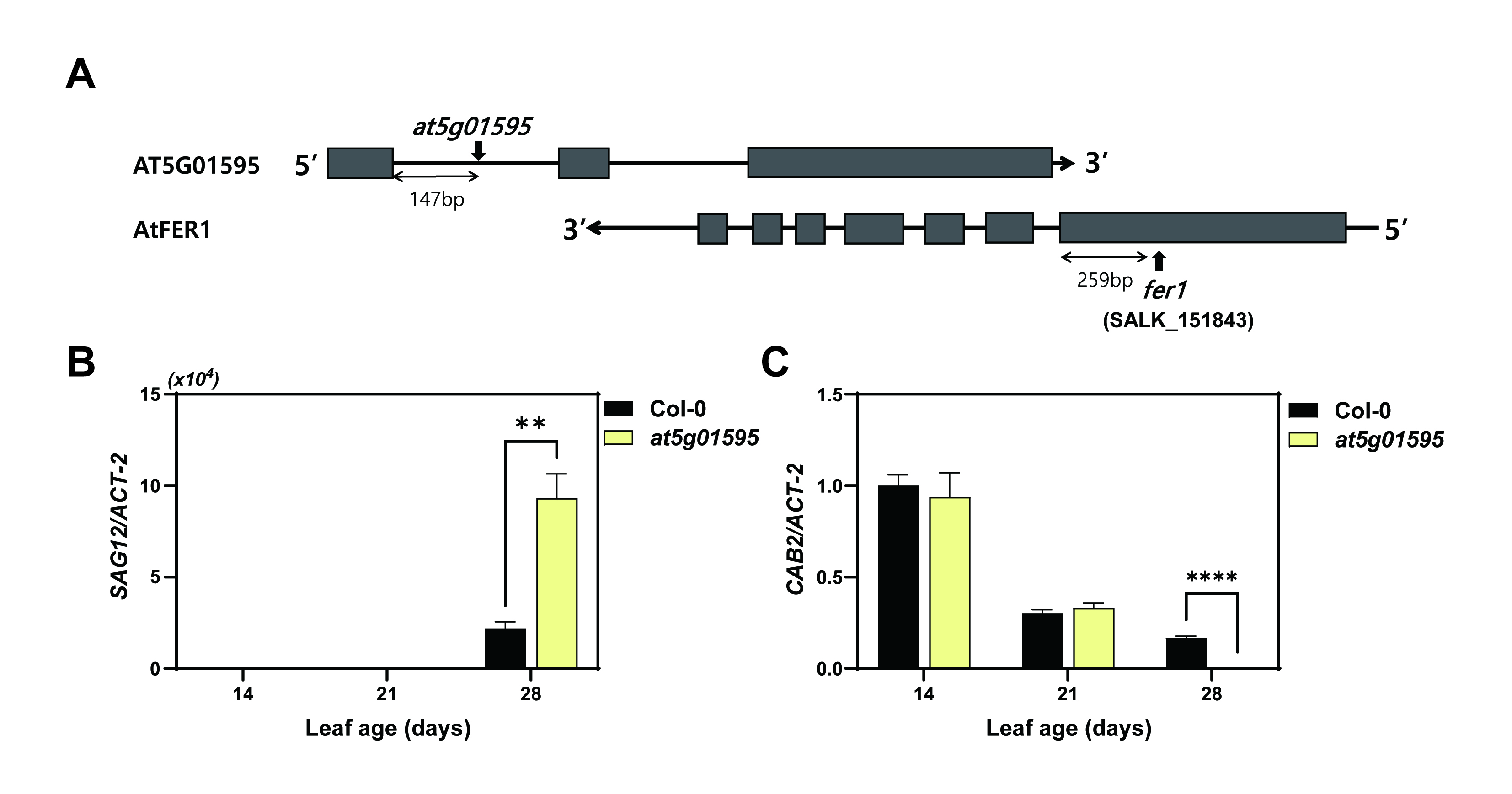

Supplement: Supplementary Figure 4 — Genomic structure of FER1 and AT5G01595 and changes in senescence-associated gene expression during developmental leaf senescence. (A) Schematic structure of FER1 and AT5G01595. The filled boxes for exons and the T-DNA insertion sites are indicated. (B, C) Senescence marker genes expression in wild-type (Col-0) and at5g01595. (B) SAG12 and (C) CAB2 expressions were detected by qRT-PCR in third and fourth leaves along the leaf age. [file Image_4.jpeg]

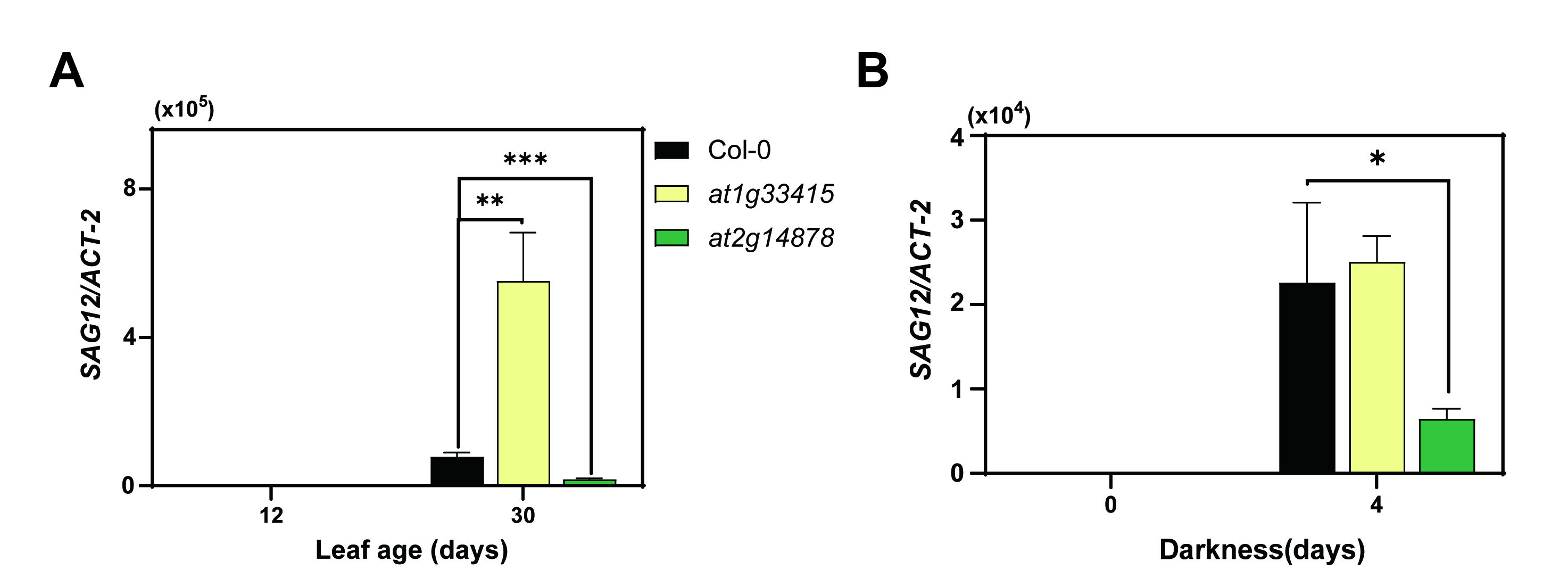

Supplement: Supplementary Figure 5 — Changes in senescence-associated gene expression in at1g33415 and at2g14878 during developmental and dark-induced leaf senescence. (A, B) Expression analysis of SAG12 in wild-type (Col-0), at1g33415 and at2g14878 leaves during developmental (A) and dark-induced leaf senescence (B) by qRT-PCR. In (A) and (B), data the mean of two replicates, and error bars represent SD (n = 2). Statistical analysis was performed using student’s t-test (*, p < 0.05; **, p < 0.01; ***, p<0.001). [file Image_5.jpeg]
